# Synthesis, Characterization and Sorption Ability of Epoxy Resin-Based Sorbents with Amine Groups

**DOI:** 10.3390/polym13234139

**Published:** 2021-11-27

**Authors:** Beata Podkościelna, Monika Wawrzkiewicz, Łukasz Klapiszewski

**Affiliations:** 1 Department of Polymer Chemistry, Institute of Chemical Sciences, Faculty of Chemistry, Maria Curie-Skłodowska University, M. Curie-Skłodowska Sq.3., PL-20031 Lublin, Poland; beata.podkoscielna@mail.umcs.pl; 2 Department of Inorganic Chemistry, Institute of Chemical Sciences, Faculty of Chemistry, Maria Curie-Skłodowska University, M. Curie-Skłodowska Sq.3., PL-20031 Lublin, Poland; monika.wawrzkiewicz@mail.umcs.pl; 3 Institute of Chemical Technology and Engineering, Faculty of Chemical Technology, Poznan University of Technology, Berdychowo 4, PL-60965 Poznań, Poland

**Keywords:** epoxy resin, TETA, crosslinking, polymeric sorbents, acid dye, textile effluents

## Abstract

Water pollution by toxic substances, such as azo dyes, is a serious environmental problem that needs to be addressed. This study presents the synthesis and characterization of new polymeric sorbents, based on the epoxy resin Epidian^®^ 5 (Ep5), as a potential adsorbent for the removal of the toxic azo dye C.I. Acid Violet 1 (AV1). Triethylenetetramine (TETA) was applied as a cross-linking agent in the amounts of 1 g (6.67 wt %), 1.5 g (10 wt %), and 2 g (13.33 wt %). The use of a compound with amino groups allows for the simultaneous functionalization of the obtained material. The reaction was carried out in an environment of ethylene glycol, with the addition of a porophore solvent (toluene) and bis(2-ethylhexyl)sulfosuccinate sodium salt (S). The attenuated total reflectance–Fourier transform infrared spectroscopy (ATR-FTIR) revealed the existence of a strong band in the 828–826 cm^−1^ range corresponding to the second-order amine group, which indicates their incorporation into the epoxy structure. The glass transition and decomposition temperatures of the resins decreased with the increasing amounts of amine in the material. The thermogravimetry (TGA) analysis demonstrated that all products are thermally stable up to 340 °C. The surface morphology and microstructural properties of the obtained sorbents were determined using scanning electron microscopy (SEM) images and showed an irregular star shape, with dimensions ranging from 400 to 1000 µm. The adsorption capacities of Ep5-TETA1, Ep5-TETA1.5, Ep5-TETA2 and Ep5-TETA1.5 + S for AV1 evaluated during batch experiments were found to be 2.92, 3.76, 7.90 and 3.30 mg/g, respectively.

## 1. Introduction

Epoxy resins (E_p_) are macromolecules with at least two epoxy groups, capable of undergoing a polyreaction that results in cross-linked and insoluble products. Epoxy resins are divided into two main categories: glycidyl resins and non-glycidyl resins. Glycidyl epoxy resins are formed by the reaction of epichlorohydrin with compounds that contain no less than two active hydrogen atoms, e.g., phenolic compounds, amino-phenols, diols and polyols, mono- and diamines [1]. Non-glycidyl epoxy resins are divided into resins with an epoxy group attached to the aromatic ring, and resins with an epoxy group bound to an aliphatic chain. They are formed during the epoxidation of unsaturated compounds with hydrogen peroxide or peracetic acid [2].

Epoxy resins are included in the group of step-growth polymerization reactions between two components. The first component is a liquid oligomer with reactive epoxide groups on the ends of the chains; the second one is the crosslinker. The oligomer (prepolymer) is formed by the reaction of a compound that includes two nucleophilic OH groups, and the other, a monomer, has C–O and C–Cl polar bonds. The nucleophile attacks the epoxy ring, which displaces the chlorine via an intermolecular SN2 reaction, forming a new epoxy group that will again react with the nucleophile to create secondary alcohol [1,3].

Since the 1960s, epoxy resins have been prepared using the reaction of bisphenol A with an excess of epichlorohydrin in an alkaline medium. The process proceeds gradually until all phenolic groups are exhausted in the ring-opening reaction. Next, the very reactive epoxide rings can be opened, for example by the nucleophilic amines, to cross-link polymeric chains, causing the polymer to become rigid and hard [4,5,6,7].

The cross-linking of epoxy resins occurs through the chemical reaction of the resin with the functional groups of an appropriately selected crosslinker. During the curing process, the linear resins are transformed into a spatially cross-linked polymer. Cross-linked resins are characterized by high mechanical strength, hardness and brittleness, high chemical resistance and electrical isolation, good adhesion to metals, glass, ceramics and wood, etc. [8,9,10,11,12,13,14].

Owing to their availability and low cost, epoxy resins are used in many fields of industry and are also used as functional sorbents. Storski et al. reported the synthesis and characterization of a new sorbent phase based on a commercial epoxy resin, for use as an alternative material in the stir-bar sorptive extraction (SBSE) technique [15]. Chopabayeva and Mukanov presented novel nano-, meso- and macroporous sorbents based on hydrolyzed lignin, synthesized by the catalytic o-alkylation of this biopolymer with epoxy resins and the subsequent amination of the formed α-oxyde derivative [16]. Novolac resin-based two-polymer networks, synthesized using novolac-based epoxy resins and low-molecular-weight amines, were studied by Ghosh and Acharyya. The synthesized materials were applied as very effective tools for the removal of azo dye molecules from an aqueous solution [17]. Hamdy et al. reported the cross-linking of polyethyleneimine by the bisphenol-A diglycidyl ether (DER) to form support-free sorbent materials. Prior to the cross-linking reaction, the polyamine chain was functionalized with hydrophobic additives. The additives affect the cross-linking and sorption efficiency [18]. Nguyen et al. prepared sizable macroporous and monolithic supports and studied their application in flow systems. The materials were obtained from epoxy resins and polyamines via emulsion polymerization. The materials characterized by maximum fluid permeability, mechanical stability, and controlled porosity were studied in detail [19]. Kurczewska and Schroeder reported the synthesis of epoxy resin modified with amine as an effective complexing agent of metal cations. Poly[(phenyl glycidyl ether)-*co*-formaldehyde] and diethylenetriamine were used as compounds in the synthesis of polymeric supports. The materials were characterized by a very good sorption capacity toward copper ions [20]. Kambarova et al. described natural minerals (zeolite) modified with polyethylene polyamine and epoxy resins. The optimal conditions for the pH sorption of lead ions under static conditions were determined [21]. The aim of this study was to use a cheap and easily available epoxy resin, Epidian^®^ 5, with triethylenetetramine as the cross-linking agent for the synthesis of functional sorbents. The proposed methodology of sorbent preparation in the form of particles, based on the epoxy resin Epidian^®^ 5 and TETA, is new and has not been previously described in the literature. The cross-linking reaction was carried out in an ethylene glycol environment, with the addition of toluene as a porophore and intensive mixing to separate the resulting particles. A water-free environment is essential because TETA mixes with water and cross-linking would not occur. The chemical structure of the obtained sorbents was confirmed by ATR-FTIR analysis. The ability to swell in selected organic solvents was also evaluated. For selected systems, thermal properties were evaluated using differential scanning calorimetry (DSC) and thermogravimetry (TG), and elemental analysis was performed to confirm the presence of nitrogen in the sorbent structure. In order to visualize the material, images were also obtained using an optical microscope and SEM. The materials were tested for their sorption ability to remove an azo acid dye from aqueous solutions.

## 2. Materials and Methods

### 2.1. Materials

Triethylenetetramine, glycol ethylene, toluene, methanol, dichloromethane and acetone were obtained from Avantor Performance Materials Poland SA (Gliwice, Poland). Bis(2-ethylhexyl)sulfosuccinate sodium salt (S) was obtained from Fluka AG (Buchs, Switzerland). The epoxy resin Epidian^®^ 5 (Ep5) was obtained from Ciech Sarzyna (Nowa Sarzyna, Poland), epoxide number 0.49–0.51 mol/100 g, viscosity at 25 °C 200–400 mPa·s.

**Figure 1:**
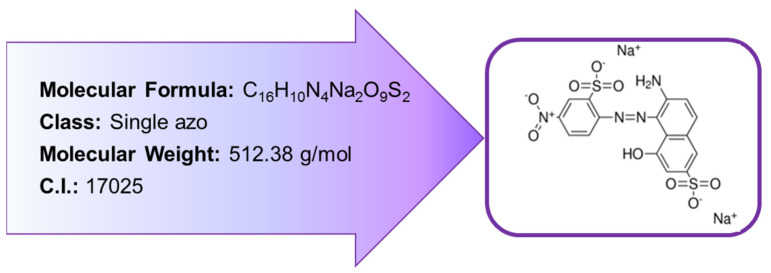
Dye characteristics.

C.I. Acid Violet 1 (AV1) dye (see Figure 1) aqueous solutions were used as adsorbates. The dye was purchased from Boruta-Kolor S.A. (Zgierz, Poland) and used without additional purification.

### 2.2. Synthesis of Polymeric Sorbents

**Figure 2:**
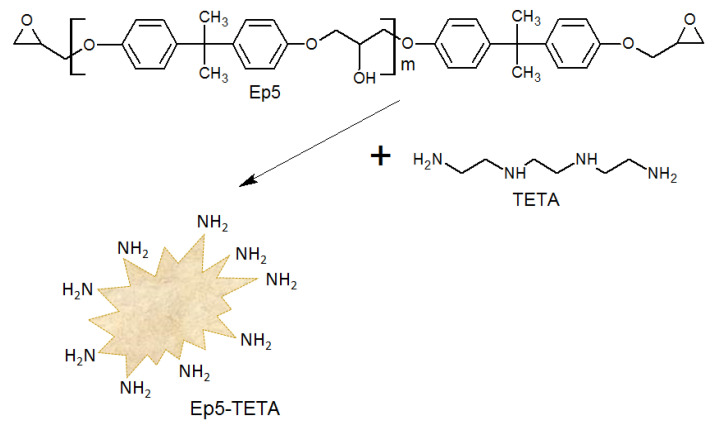
Chemical structures of reagents and a simplified synthesis scheme.

Ethylene glycol (150 mL) was added to a 250 mL round-bottomed flask, fitted with a mechanical stirrer and thermometer. Epidian^®^ 5 resin, triethylenetetramine and toluene were mixed in a separate vessel. The prepared mixture was added into the flask with ethylene glycol during stirring. Polymerization was carried out for 12 h with intensive stirring (300/min), initially at room temperature, and was gradually increased to 100 °C. The syntheses were carried out in non-water, ethylene glycol medium, with one sample (Ep5-TETA1.5 + S) also having the addition of surfactant: di(2-ethylhexyl)sulfosuccinate sodium salt [22]. The chemical structures of Ep5 and TETA are presented in Figure 2.

Detailed information regarding reagents and their amounts is presented in Table 1. Additionally, the results of the elemental analysis confirmed the participation of TETA in the sorbent structure.

### 2.3. Characteristics of Polymeric Sorbents

The surface morphology and microstructural properties of the obtained samples were determined, based on SEM images obtained using a Tescan VEGA3 scanning electron microscope (Tescan Orsay Holding a.s., Brno, Czech Republic). Furthermore, the images of the polymeric sorbents were obtained by using a Malvern optical microscope (Malvern, Great Britain).

The CHN elemental analysis was carried out using a 2400 Perkin-Elmer Inc. apparatus (Waltham, MA, USA). A 10 mg sample was placed in a tin capsule, and all results are given as an average of three measurements.

The attenuated total reflection (ATR) was recorded based on Fourier transform infrared spectroscopy (ATR-FTIR) using a TENSOR 27 Bruker spectrometer equipped with a diamond crystal (Ettlingen, Germany). The spectra were recorded in the range of 4000–600 cm^−1^ with 32 scans per spectrum, at a resolution of 4 cm^−1^.

Differential scanning calorimetry (DSC) curves were obtained with the use of a DSC Netzsch 204 calorimeter Netzsch (Günzbung, Germany). The measurements were taken in aluminum pans with a pierced lid. The sample mass was approx. 10 mg under a nitrogen atmosphere (30 cm^3^/min). Dynamic scans were performed at a heating rate of 10 °C/min in the temperature range of 0–200 °C. Additionally, in order to evaluate the T_g_ (glass transition temperature), a heating rate of 2 °C/min in the temperature range of −15 to 30 °C was applied. An empty aluminum crucible was used as a reference.

Thermogravimetric analysis TG/DTG was conducted using an STA 449 Jupiter F1, Netzsch (Selb, Germany). The samples were heated from 25 to 1000 °C at a rate of 10 °C/min in a dynamic atmosphere of helium (25 cm^3^/min). An empty Al_2_O_3_ crucible was used as a reference. The thermal stability factors, such as mass loss temperatures (*T*_5%_, *T*_10%_, *T*_50%_), as well as temperatures of maximum mass loss (*T*_max_) and residual mass (*RM*) were estimated.

### 2.4. Adsorption Tests

The adsorption experiments were performed using the batch mode method at room temperature. A weighted amount of the resin (0.02 g) was shaken using an Elpin Plus laboratory shaker (Lubawa, Poland) with 20 mL of the AV1 solution at a strictly defined initial concentration (*C*_0_ = 10–500 mg/L). The initial pH of the solutions was ~4.85. After a predetermined phase contact time (t), the resin was separated by filtration. The AV1 content in the filtrate was measured spectrophotometrically at λ_max_ = 552 nm using a Cary 60 UV–VIS spectrophotometer (Agilent Technologies, Santa Clara, CA, USA).

The adsorption capacity (*q_e_*) (at *t* = 24 h) was calculated based on Equation (1):

(1) $$q_{e}=\frac{\left(C_{0}-C_{e}\right)}{m}V$$

where *C*_0_—AV1 initial concentration (mg/L), *C_e_*—AV1 concentration at equilibrium (mg/L), *V*—volume (L), *m*—resin mass (g).

The Langmuir, Freundlich and Dubinin–Radushkevich adsorption isotherms were used to determine the balance between the AV1 concentration in the resin solid phase and its concentration in the liquid phase at equilibrium (Equations (2)–(4)) [23,24,25]:

(2) $$\frac{C_{e}}{q_{e}}=\frac{1}{Q_{0}b}+\frac{C_{e}}{Q_{0}}\;$$

(3) $$logq_{e}=logk_{F}+\frac{1}{n}logC_{e}\;$$

(4) $$lnq_{e}=lnq_{m}-k_{DR}\varepsilon^{2}\;$$

where: *C_e_*—AV1 concentration at equilibrium (mg/L), *Q*_0_—monolayer capacity (mg/g), *b*—the Langmuir constant (L/mg), *q_e_*—adsorption capacity (mg/g), *k_F_*—the Freundlich constant (mg^1−1/n^·L^1/n^/g), 1/n—parameter characterizing the energy heterogeneity of the adsorbent surface, *q_m_*—maximum adsorption capacity (mg/g), *k_DR_*—constant related to the adsorption energy (mol^2^/J^2^), *ε*—adsorption potential (J/mol) (calculated as $\varepsilon =RTln\left(1+\frac{1}{C_{e}}\right)$ where *R* is the gas constant 8.314 J/mol·K, and *T* is the temperature (K)).

## 3. Results and Discussion

### 3.1. Visualization of Sorbents

**Figure 3:**
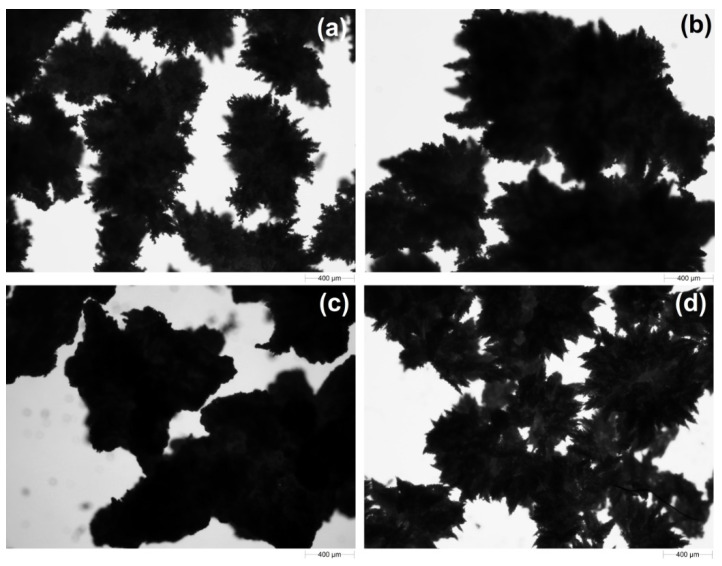
Optical images of obtained sorbents: (**a**) Ep5-TETA1, (**b**) Ep5-TETA1.5, (**c**) Ep5-TETA2 and (**d**) Ep5-TETA1.5 + S.

**Figure 4:**
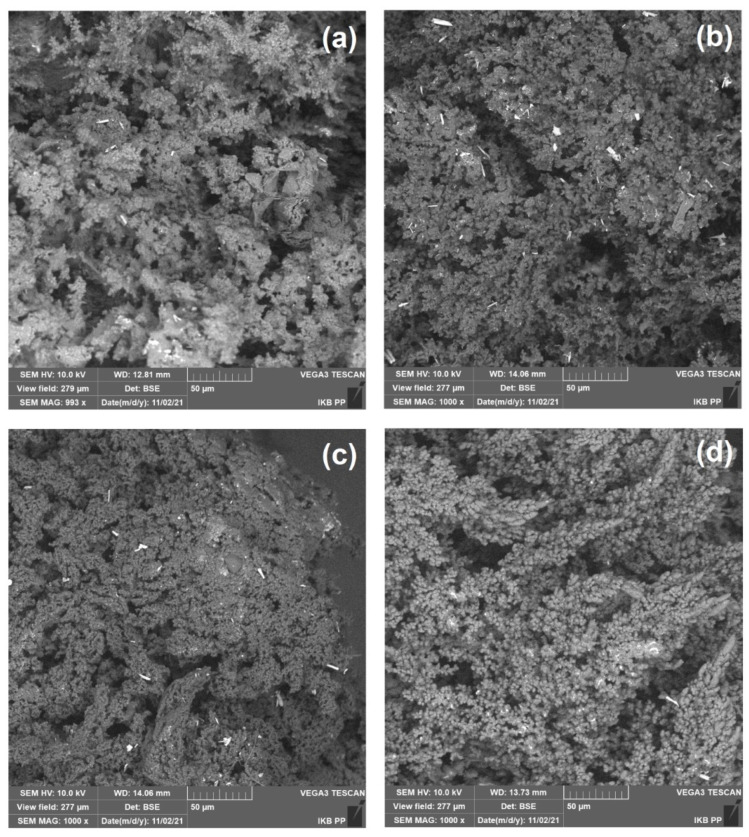
SEM images of obtained sorbents: (**a**) Ep5-TETA1, (**b**) Ep5-TETA1.5, (**c**) Ep5-TETA2 and (**d**) Ep5-TETA1.5 + S.

The images of the synthesized polymeric sorbents using an optical microscope are presented in Figure 3. Moreover, SEM pictures of these materials are presented in Figure 4. As can be seen, the sorbents are characterized by an irregular star shape. With an increase in the amount of crosslinking agent, less-branched materials are received. The particle size ranges from 400 to 1000 µm; as the cross-linking of the material increases, an increase in its size is observed. The addition of a surfactant has a positive effect on particle size and the level of separation.

### 3.2. ATR-FTIR Analysis

**Figure 5:**
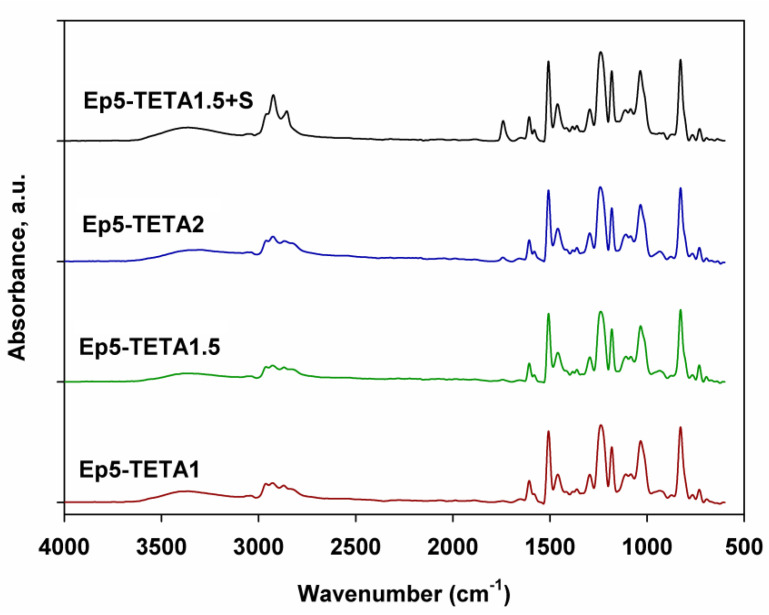
ATR-FTIR spectra of obtained polymeric sorbents.

In the ATR-FTIR spectra of all the obtained adsorbents (see Figure 5), the band in the range of 3395–3310 cm^−1^ originates from the stretching vibration of the second-order amine group and the hydroxyl group. Conversely, in the 2963–2854 cm^−1^ region, symmetric and asymmetric stretching vibrations of the methyl and methylene groups were observed [26]. The doublet in the range of 1508–1458 cm^−1^ indicates the presence of a stretching vibration of the aromatic ring. In the 1295–1181 cm^−1^ region, there is a stretching vibration of the C-N bond. The vibration originating from the carbon–oxygen bonding of the hydroxyl group can be observed in the range of 1037–1032 cm^−1^. A strong band in the 828–826 cm^−1^ region indicates the presence of the deformation vibration of the second-order amine group.

In the presented spectra, a very weak signal at approx. 915 cm^−1^, corresponding to the presence of epoxy groups, can be observed, which indicates the cross-linking of the epoxide with amines [26].

### 3.3. DSC Analysis

**Figure 6:**
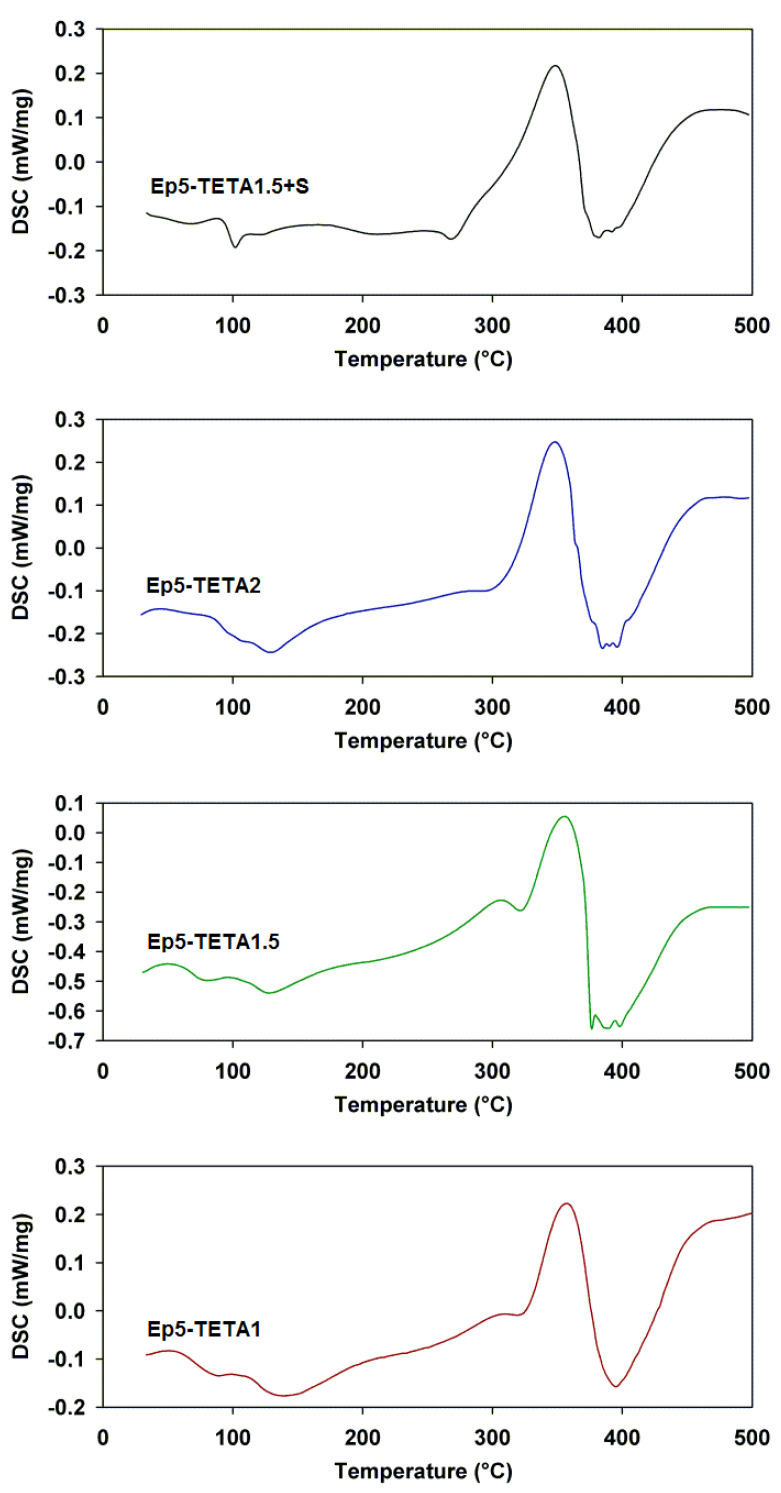
DSC curves of obtained polymeric sorbents.

The DSC method was used to determine the thermal properties of sorbents with different contents of triethylenetetramine and the addition of di(2-ethylhexyl) sulfosuccinate sodium salt (see Figure 6). Characteristic parameters, such as glass transition temperature (*T*_g_) and maximum peak temperature (*T*_max_), as well as initial (*T*_onset_) and final (*T*_offset_) peak temperatures, were determined. These values are summarized in Table 2.

**Figure 7:**
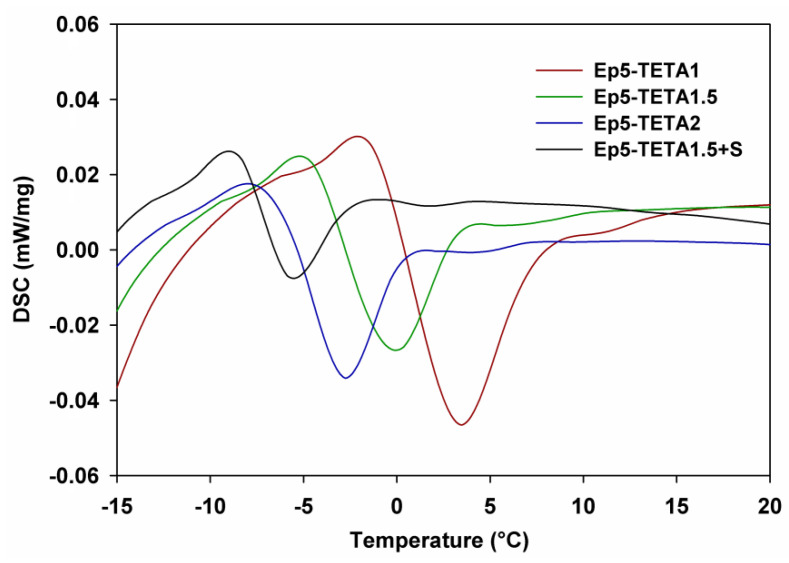
DSC curves in the region of glass transition temperatures.

All DSC curves show two endothermic effects and one exothermic effect. The glass transition temperatures (*T*_g_) of the materials are noticeable in the range of 27–38 °C. The endothermic effect that occurs at temperatures between 102 and 138 °C is associated with the evaporation of water and unreacted amine. The exothermic peak (350 °C) most likely originates from reactions between the amine and the uncrosslinked epoxy groups. The intense endoenergetic effect comes from the thermal decomposition of the sorbents. The decomposition temperatures are in the range of 267–393 °C, with a peak maximum (*T*_max_) of 348–358 °C. The glass transition and decomposition temperatures decrease with the increasing amount of amine in the material. In Figure 7, the glass transition temperature for studied samples is presented. The measurements were performed in the following range of temperatures: from −15 to 30 °C. As can be observed, the *T*_g_ decreases with the addition of TETA (from 3.2 to −3.1 °C). The lowest temperature was obtained for the sample with the addition of surfactant.

### 3.4. TGA Analysis

**Figure 8:**
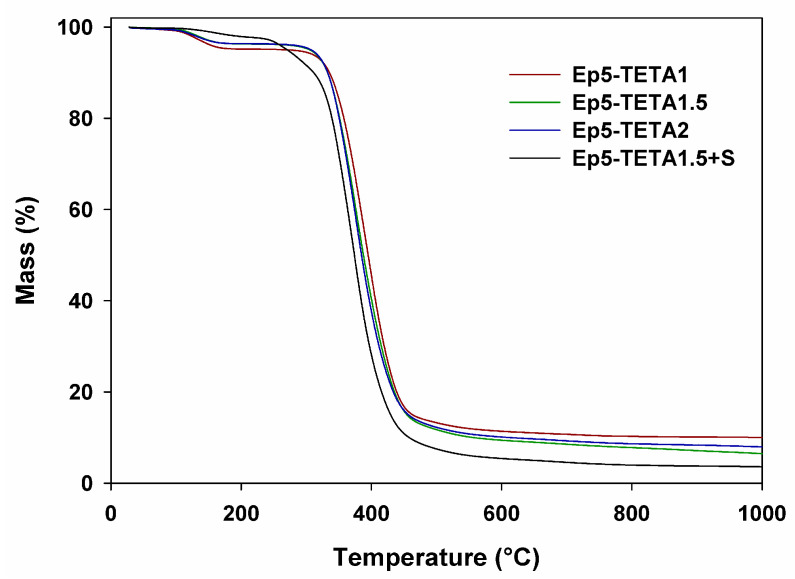
TGA curves of obtained polymeric sorbents.

The thermal stability and degradation behavior of the obtained products were investigated by means of thermogravimetry. The TGA results of the thermal decomposition process in the inert atmosphere of helium are presented in Figure 8 and Table 3.

Based on the analysis of the thermogravimetric curves and the data summarized in Table 3, it can be concluded that with the increasing amount of TETA, the polymer sorbents are more thermally stable, which is confirmed by their better crosslinking. All products are thermally stable up to a temperature of 340 °C, after which they decompose in one step in the temperature range of 340–420 °C. Moreover, on the basis of the analyzed curves, it can be seen that the addition of a surfactant has a positive effect on thermal stability (especially in the initial heating phase). A slight loss of mass of the products, related to the physically adsorbed water on the surface of polymer composites, which can be observed in the temperature range of 100–200 °C, is also noteworthy.

### 3.5. Adsorption Tests

**Figure 9:**
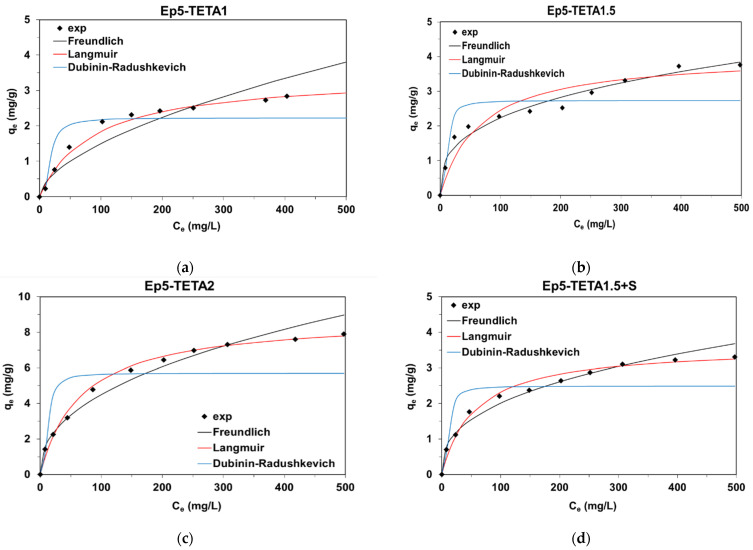
Equilibrium data and the fitting of experimental points to the Langmuir, Freundlich and Dubinin–Radushkevich models for AV1 adsorption on (**a**) Ep5-TETA1, (**b**) Ep5-TETA1.5, (**c**) Ep5-TETA2 and (**d**) Ep5-TETA1.5+S.

Adsorption studies conducted at equilibrium allow for the determination of the sorption capacity. The experimental values of the adsorption capacities regarding AV1 dye, as determined based on Figure 9, were equal to 2.92, 3.76, 7.90, and 3.30 mg/g for Ep5-TETA1, Ep5-TETA1.5, Ep5-TETA2, and Ep5-TETA1.5+S, respectively. These values increased with the increasing functional group (TETA) content in the resins.

The experimental data were fitted to the three popular isotherm models, namely, the Langmuir (Equation (2)), Freundlich (Equation (3)) and Dubinin–Radushkevich (Equation (4)) isotherms, in order to determine the mechanism of AV1 uptake by the epoxy resins. The parameters under discussion are listed in Table 4.

Taking into consideration the values of the determination coefficients *R*^2^, it can be stated that the Langmuir model, which considers the formation of a monolayer coverage of dye molecules on the resins’ surface, seems to be the best one. The *R*^2^ values were in the range from 0.989 to 0.996. The calculated monolayer adsorption capacities *Q*_0_ were equal to 3.44 mg/g for Ep5-TETA1, 4.06 mg/g for Ep5-TETA1.5, 8.83 mg/g for Ep5-TETA2 and 3.61 mg/g for Ep5-TETA1.5+S. The Langmuir constant *k_L_* increased from 0.012 to 0.018 L/mg with the increasing amount of TETA.

**Figure 10:**
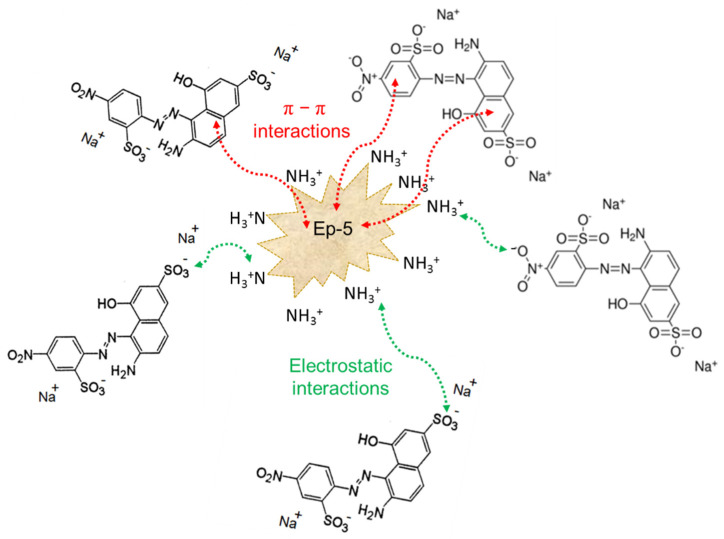
Probable mechanism of interactions between AV1 dye and the functionalized epoxy resins in acidic media.

Lower values of the determination coefficients (*R*^2^ = 0.886−0.980) characterized the fit of the experimental data to the Freundlich model, which assumes a multilayer adsorption mechanism of the adsorbate on the surface of polymer resins. The Freundlich constants *k_F_* for the TETA functionalized epoxy resins ranged from 0.104 to 0.621 mg^1−1/n^·L^1/n^/g, while the parameters characterizing the energy heterogeneity of the adsorbent surface, 1/n, were lower than 1, indicating favorable adsorption of AV1 of a physical nature, involving interactions between positively charged amine functionalities with negatively charged sulphonic groups that are present in the dye anions. The adsorption mechanism may also involve weak π–π interactions between the resin backbone, containing aromatic rings, and the benzene rings that are present in the dye structure. Possible interactions in the adsorption system under acidic conditions are presented in Figure 10.

The values of theoretical monolayer capacities *q_m_*, as determined based on the Dubinin-Radushkevich model, were lower (*q_m_* = 2.23−5.70 mg/g) than the experimental results (2.92–7.90 mg/g) corresponding to the adsorption isotherm plateau, which indicates that the modeling of the Dubinin–Radushkevich scheme for the adsorption system is unacceptable. This is confirmed by the values of the determination coefficients, ranging between 0.651 and 0.854. The mean free energy ($E=\frac{1}{\sqrt{2k_{DR}}}$) for AV1 removal from its adsorption site to the infinity was calculated and is equal to approximately 3.65 kJ/mol for the investigated adsorption systems.

The adsorption abilities of the investigated polymers were compared with the available literature data. Unfortunately, not much information can be found regarding the adsorptive removal of this dye. According to Namasivayam et al. [27], AV1 adsorption on waste red mud, originating from bauxite processing, can be described using both the Langmuir and Freundlich models. The adsorption capacity of red mud for AV1 was calculated as 1.37 mg/g at pH 4.1. The AV1 uptake by red mud included electrostatic attraction and ion exchange. Although this adsorbent did not show a high sorption capacity toward AV1, its undoubted advantage was its price, as it is a waste product. The polystyrene anion exchangers with quaternary ammonium groups, such as Purolite A520E and Lewatit S5428, are characterized by significant uptake (≈ 835 mg/g) of AV1 but, unfortunately, they are adsorbents with much higher operating costs [28]. It was previously evaluated that divinylbenzene copolymer with glycidyl methacrylate, functionalized with TETA, is able to adsorb 172 mg of AV1 per 1 g and physical and chemical interactions are responsible for dye-binding [29].

## 4. Conclusions

As part of this work, a cheap and easily available epoxy resin, Epidian^®^ 5, with triethylenetetramine as the cross-linking agent was used for the synthesis of functional sorbents. The cross-linking reaction was carried out in an ethylene glycol environment with the addition of toluene as a porophore and intensive mixing to separate the resulting particles. A water-free environment is essential because TETA mixes with water and cross-linking would not occur. The highest nitrogen content values of 4.396 and 4.327 were observed when 2 g and 1.5 g of the crosslinking agent were added. The surfactant content (sample Ep5-TETA1.5+S) had a very positive effect on the incorporation of TETA into the structure of the formed polymer network.

The ATR-FTIR analysis confirmed the presence of amine groups in the synthesized materials. The stretching vibrations of the C–N bond (1295–1181 cm^−1^) and deformation vibrations of the second-order amine group (828–826 cm^−1^) are visible in the spectra. DSC curves show two endothermic and one exothermic effects. The exothermic effect (~350 °C) probably originates from reactions between the amine and free epoxy groups. The decomposition temperatures are in the range of 267–393 °C, with a peak maximum at 348–358 °C. Thermogravimetric analysis confirms these observations. All polymeric sorbents are thermally stable up to a temperature of 340 °C. Moreover, it was concluded that the addition of a surfactant had a positive effect on thermal stability (especially in the initial heating phase).

Adsorption tests confirmed that epoxy polymers, functionalized with TETA, can be used as adsorbents for the removal of azo dyes, such as AV1. It was shown that as the nitrogen content in the adsorbents increases, their sorption capacity toward AV1 increases. The values of sorption capacities as determined experimentally ranged from 2.92 to 7.90 mg/g, and the Langmuir model described the adsorption system at equilibrium, as evidenced by the values of the determination coefficients (*R*^2^ = 0.989 − 0.996). The values of monolayer capacities calculated based on the Langmuir model were in the range of 3.44–8.93 mg/g. The adsorption studies carried out may be of great cognitive interest for the development of effective adsorption materials that can be used in textile wastewater treatment technologies regarding various types of azo dyes.

## Figures and Tables

**Table 1:** Experimental parameters of the synthesis.

Sample	Ethylene Glycol (mL)	Epidian 5 (g)	TETA (g)/(wt %)	Toluene (mL)	Surfactant (g)	N (%)
Ep5-TETA1	150	15	1.0/(6.67)	10	-	2.774
Ep5-TETA1.5			1.5/(10.00)		-	3.523
Ep5-TETA2			2.0/(13.33)		-	4.396
Ep5-TETA1.5+S			1.5/(10.00)		1.5	4.327

**Table 2:** DSC data of obtained polymeric sorbents.

Samples	*T*_g_ (°C)	*T*_onset_ (°C)	*T*_max_ (°C)	*T*_offset_ (°C)
Ep5-TETA1	3.2	320	358	393
Ep5-TETA1.5	−0.5	322	356	375
Ep5-TETA2	−3.1	294	348	384
Ep5-TETA1.5+S	−5.5	267	349	382

**Table 3:** TGA data of obtained polymeric sorbents.

Samples	*T*_1%_ (°C)	*T*_5%_ (°C)	*T*_50%_ (°C)	*RM* (%)
Ep5-TETA1	105	271	395	10.0
Ep5-TETA1.5	118	305	384	6.0
Ep5-TETA2	119	309	385	7.5
Ep5-TETA1.5+S	151	270	373	3.4

Note: *T*_1%_, *T*_5%_ and *T*_50%_—the temperature of 1%, 5%, and 50% mass loss from the TGA curve, respectively; RM—residual mass at 1000 °C.

**Table 4:** The parameters of the Langmuir, Freundlich and Dubinin–Radushkevich isotherms for AV1 adsorption on the epoxy resins.

Parameters	Ep5-TETA1	Ep5-TETA1.5	Ep5-TETA2	Ep5-TETA1.5+S
Langmuir				
*Q*_0_ (mg/g)	3.44	4.06	8.83	3.61
*k_L_* (L/mg)	0.012	0.015	0.018	0.015
*R* ^2^	0.989	0.963	0.996	0.993
Freundlich				
*k_F_* (mg^1−1/n^·L^1/n^/g)	0.104	0.475	0.621	0.353
1/n	0.579	0.337	0.430	0.378
*R* ^2^	0.886	0.937	0.980	0.973
Dubinin-Radushkevich				
*k_DR_* (mol^2^/J^2^)	3.69 × 10^−5^	1.61 × 10^−5^	1.89 × 10^−5^	1.70 × 10^−5^
*q_m_* (mg/g)	2.23	2.73	5.70	2.49
*R* ^2^	0.854	0.759	0.651	0.696
